# Headache and Papilledema Unmasking an Underlying Myeloproliferative Neoplasm: A Case of Chronic Recanalized Cerebral Venous Sinus Thrombosis

**DOI:** 10.7759/cureus.101051

**Published:** 2026-01-07

**Authors:** Shamera Hossain, Tameem Tawfiq

**Affiliations:** 1 Acute Medicine, Bradford Teaching Hospitals NHS Foundation Trust, Bradford, GBR

**Keywords:** cerebral venous sinus thrombosis (cvst), idiopathic intracranial hypertension (iih), jak2 mutation, myeloproliferative neoplasm (mpn), recanalized thrombus

## Abstract

Cerebral venous sinus thrombosis (CVST) is a potentially life-threatening condition that can present with clinical features mimicking idiopathic intracranial hypertension (IIH), as both may present with headache and bilateral papilledema, leading to diagnostic uncertainty. We report a case of a 50-year-old woman referred for suspected IIH in whom venous neuroimaging revealed chronic, partially recanalized CVST. Thrombocytosis on full blood count, previously uninvestigated, prompted hematological assessment and led to the diagnosis of a JAK2-positive myeloproliferative neoplasm, for which cytoreductive therapy was initiated alongside anticoagulation. This case underscores the importance of performing venous imaging in patients with headache and papilledema, even when features suggest IIH, and highlights that thrombocytosis should prompt evaluation for an underlying myeloproliferative disorder to guide long-term management and prevent recurrent thrombosis.

## Introduction

Cerebral venous sinus thrombosis (CVST) is a rare but potentially life-threatening cerebrovascular disorder that occurs more commonly in young adults [1]. Thrombosis may develop within the dural venous sinuses or one or more cerebral veins, leading to impaired venous drainage and increased intracranial pressure. Patients may present with nonspecific symptoms such as headache, vomiting, dizziness, seizures, or altered consciousness.

The risk factors for CVST are broadly divided into congenital and acquired causes. Congenital or genetic risk factors include inherited thrombophilias, such as the Factor V Leiden mutation, and deficiencies of protein C or protein S. Acquired risk factors include pregnancy, postpartum period, head trauma, infection, and recent surgery [2].

Thrombocytosis is a well-recognized risk factor for thromboembolic events. It is defined as a platelet count above 450 × 10^9^/L and can be classified as primary or secondary [3]. Primary thrombocytosis results from clonal proliferation of hematopoietic stem cells, as seen in myeloproliferative neoplasms (MPNs) such as essential thrombocythemia. Secondary or reactive thrombocytosis occurs in association with chronic inflammation, infection, malignancy, iron deficiency, or post-splenectomy states. While reactive thrombocytosis resolves with management of the underlying cause, primary thrombocytosis may predispose to both thrombosis and bleeding complications [3].

Idiopathic intracranial hypertension (IIH) is characterized by features of raised intracranial pressure in the absence of hydrocephalus, mass lesion, or abnormal cerebrospinal fluid composition [4]. CVST can mimic IIH, as both conditions share similar clinical manifestations, including headache, visual blurring, tinnitus, and diplopia. However, in cases of chronic or recanalized CVST, symptoms may persist despite partial restoration of venous flow, posing a diagnostic challenge.

We present a case of a middle-aged woman who initially presented with symptoms suggestive of IIH but was ultimately found to have chronic recanalized CVST secondary to JAK2-positive myeloproliferative disease, highlighting the importance of thorough evaluation in patients with raised intracranial pressure.

## Case presentation

A 50-year-old woman was referred by the ophthalmology department following the incidental finding of grade 1 bilateral papilledema in the context of a history of headache. On further review, she reported a long-standing history of headaches, including one episode of loss of consciousness approximately five years earlier. This episode had been associated with nausea, and raised platelet counts had been noted since that time. She had initially been diagnosed with cluster headaches in 2021. In 2023, she sustained a head injury, after which her headaches, light-headedness, and tinnitus recurred. She was treated with various analgesics without significant improvement. For the six weeks prior to presentation, she had experienced continuous right-sided headache, particularly in the mornings, with associated periorbital pain that worsened on bending forward. A CT scan of the head was unremarkable. Her general practitioner referred her to the Minor Eye Conditions Service (MECS) and subsequently to ophthalmology, where papilledema was detected.

Ophthalmologic examination showed a normal optical coherence tomography (OCT) macula bilaterally. OCT of the optic disc and retinal nerve fiber layer revealed increased thickness (301 µm in the right eye and 288 µm in the left eye). Bilateral intraocular pressure was elevated (27-29 mmHg). The findings were suggestive of grade 1 papilledema, raising suspicion of IIH, with associated bilateral ocular hypertension.

**Figure 1:**
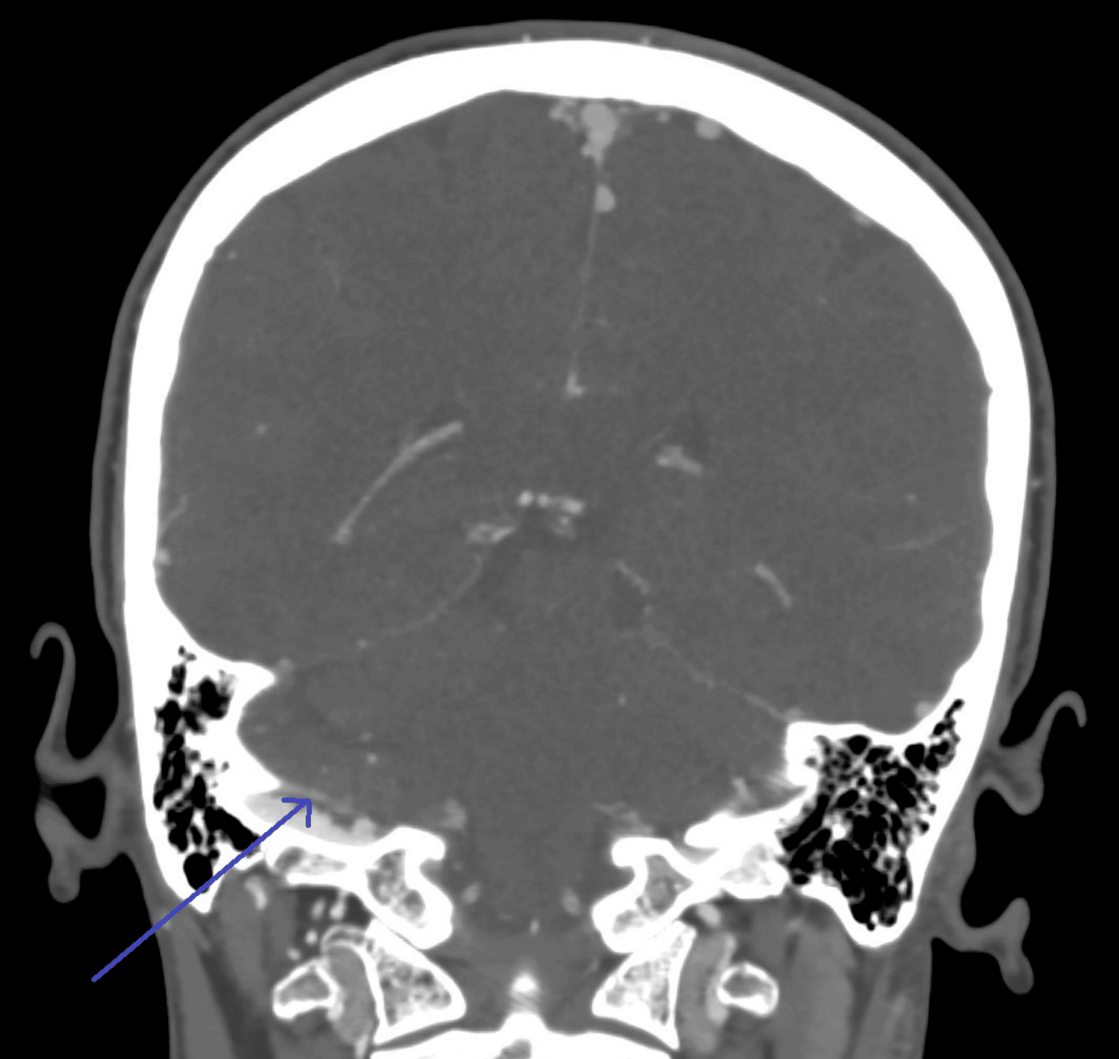
CT venogram showing a linear, eccentric thrombus against the superior wall of the right sigmoid sinus. Thin linear filling defects are present in the bilateral sigmoid sinuses, possibly representing chronic sinus thrombosis. The blue arrow indicates the linear eccentric thrombus.

Similarly, CT venography (Figure 1) showed a linear, eccentric thrombus along the superior wall of the right sigmoid sinus.

**Figure 2:**
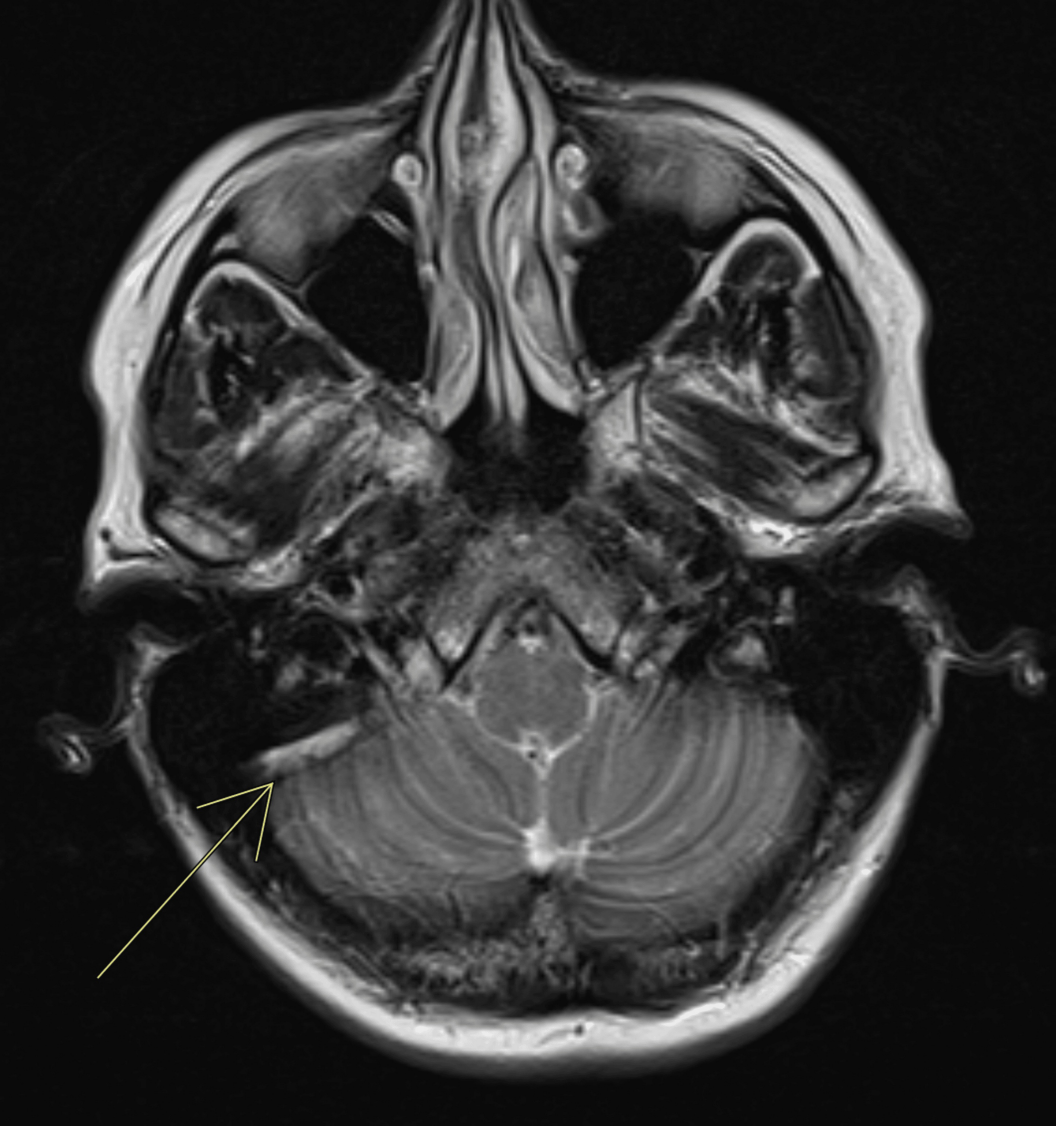
MRI head with contrast (axial T2) at the level of sigmoid sinus showing a chronic thrombus (linear hyperintensity). The white arrow indicates the chronic thrombus.

MRI head with contrast (axial T2) at the level of the sigmoid sinus showed a chronic thrombus (Figure 2).

Routine laboratory investigations revealed persistent thrombocytosis. Further hematological evaluation was undertaken to exclude secondary causes, including iron deficiency, and to assess for primary causes through flow cytometry, paroxysmal nocturnal hemoglobinuria screening, and mutational analysis for JAK2, CALR, and MPL genes. The JAK2 mutation was detected, confirming an underlying myeloproliferative neoplasm (Table 1).

**Table 1:** Laboratory test results over time. Hb: Hemoglobin; WBC: White blood cells; PLT: Platelets; TIBC: Total iron binding capacity; TSAT: Transferrin saturation; CRP: C-reactive protein; LDH: Lactate dehydrogenase; INR: International normalized ratio

Investigation (units)	Reference range	Patient values		
		26-09-2025	13-10-2025	3-12-2025 (after treatment)
Hb (g/L)	120-160	134	148	133
WBC (× 10^9^/L)	4-11	7.54	12.28	4.13
PLT (× 10^9^/L)	150-400	796	796	367
Blood film	—	Thrombocytosis	Thrombocytosis	—
Prothrombin time (s)	9.5-12.5	11.2	11	—
INR	0.8-1.2	1	0.9	—
Serum iron (umol/L)	10-30	16.1	—	—
TIBC (ug/dL)	240-450	61.3	—	—
%TSAT	20-50%	26%	—	—
Ferritin (ng/mL)	15-150	205	—	—
Sodium (mmol/L)	135-145	138	138	137
Potassium (mmol/L)	3.5-4.5	4.6	4.7	4.8
CRP (mg/dl)	<1	—	2.6	—
LDH (U/L)	140-280	—	331	—
Uric acid (mg/dL)	2.5-6.0	—	2.6	—

Lumbar puncture demonstrated an opening pressure of 29 mmHg, with normal cerebrospinal fluid composition. On examination, the patient was alert and oriented, with intact cranial nerves and no focal neurological deficits. There were no cerebellar signs, and gait was normal. Fundoscopic examination confirmed grade 1 bilateral papilledema with preserved visual acuity and visual fields.

Based on these findings, she was commenced on low-molecular-weight heparin following discussion with the hematology team and was subsequently started on cytoreductive therapy after the diagnosis of a myeloproliferative neoplasm. She remained under ongoing hematology follow-up, with improvement in headache symptoms, platelet counts, and stable vision.

## Discussion

CVST is a rare manifestation of venous thromboembolism (VTE), as the cerebral venous system is an uncommon site of thrombosis. Although its risk factors overlap with those of other thrombotic disorders, the presentation and consequences can be distinct. Anticoagulation remains the mainstay of treatment, aiming to prevent thrombus propagation and promote recanalization. CVST typically affects young adults, with a median age of approximately 35 years, and is more common in women [5]. Reported risk factors include pregnancy, postpartum period, oral contraceptive use, thrombophilia, inflammatory disease, infection, malignancy, dehydration, and head trauma [5]. Due to its unusual location, CVST warrants thorough investigation to identify the underlying precipitating factors and guide long-term management [6]

This patient presented with chronic headache and grade 1 bilateral papilledema, a presentation often initially considered as IIH. The clinical and radiological similarities between chronic or recanalized CVST and IIH can make differentiation challenging. Studies suggest that up to 10% of presumed IIH cases have an underlying CVST, though reports of chronic CVST presenting this way remain rare [7]. Recognition is important, as treatment with anticoagulation differs from standard IIH management. The presence of thrombocytosis on laboratory testing should prompt further evaluation, particularly when thrombosis occurs at an atypical site such as the cerebral venous sinuses.

A study reported that a subset of patients presumed to have IIH were later found to have CVST after detailed neuroimaging, highlighting the risk of misdiagnosis and potential for serious complications such as stroke or death [7]. Identifying the underlying cause of CVST is essential for preventing recurrence. In this patient, persistent thrombocytosis - unexplained by reactive causes - led to the discovery of an underlying JAK2-positive myeloproliferative neoplasm.

This case emphasizes the importance of maintaining a high index of suspicion for CVST in patients presenting with raised intracranial pressure and papilledema. Venous imaging should be performed in all patients before diagnosing IIH. Furthermore, persistent thrombocytosis should prompt evaluation for an underlying myeloproliferative disorder to enable early treatment and reduce the risk of recurrent thrombosis [8].

## Conclusions

This case highlights the diagnostic overlap between IIH and chronic recanalized CVST. In patients presenting with features of raised intracranial pressure, including headache and papilledema, neuroimaging with venous studies is essential to exclude CVST, even when symptoms appear chronic or mild. The presence of persistent thrombocytosis should alert clinicians to the possibility of an underlying myeloproliferative neoplasm, particularly when thrombosis occurs at an unusual site. Even partially recanalized CVST can continue to produce symptoms, highlighting the importance of recognizing both the vascular and hematological components early to guide treatment, prevent recurrence, and reduce long-term complications.
